# Review of Drs. Gardette and Trenor, on Dental Colleges and Dental Education

**Published:** 1853-01

**Authors:** W. W. H. Thackston


### ARTICLE III.

*Review of Drs. Gardette and Trenor, on Dental Colleges and Dental Education.* By W. W. H. THACKSTON, M. D., D. D. S.

AS a practicing dental surgeon, feeling a lively interest in everything calculated to effect, either beneficially or injuriously, the profession of our choice, and being an alumnus of the Baltimore College of Dental Surgery, our attention has very naturally been attracted by an attack, through the medical press, upon the policy of separate schools for the specialty of dental surgery, and a recommendation of a lectureship in the medical schools of the country as a substitute and improvement upon the present established system of dental education.

We have been struck with the ill-disguised bitterness and rancor with which, in the discussion of this subject, the Baltimore College and its faculty have been assailed. The merits of this institution, and its claims to public confidence and support, have been pointedly questioned, or totally denied; its facilities and appliances for a thorough system of instruction in dental science disparaged and derided, and its alumni held up to reproach and ridicule.

This movement has been begun, as most readers of the Journal are advised, by a dentist of Philadelphia, and advocated by a dentist of New York—individuals, if we have been correctly informed, of some local reputation as practitioners, but unfortunately for a proposition which possesses no intrinsic merit, and is dependent for its strength, upon the character and influence of its projector and advocate, their names are unheralded by any important demonstration in the cause of science, or by any valuable contribution to the common fund of knowledge. And we take the liberty, in the outset, of saying to Drs. Gardette and Trenor, that however exalted an opinion they may entertain of themselves, and for each other, and however poorly and contemptuously they may think and speak of others, science cannot yet afford to recognize either of them as oracles; and they need not grow spiteful and acrimonious when their errors are pointed out, and their follies are exposed and rebuked. Dental surgery pays no undeserved tribute to personal ambition, and humanity can ill afford sacrifices upon the altar of personal vanity.

As Drs. Gardette and Trenor have both assumed the same positions, as each appears ready and anxious to endorse the other, and as both are laboring to accomplish the same object, we have thought proper to embrace them both in one review. And while we perceive no necessity, and feel no inclination to follow each through the tortuous and labyrinthine windings of their several articles, we shall state fairly their propositions and arguments, and make such reflections as our acquaintance with the subject, and truth and justice may seem to demand.

Drs. Gardette and Trenor have assumed that the present system of dental education is radically defective; that the experiment of dental colleges has been fully and fairly tried, and has most emphatically failed; that this failure is attributable, first, to the impolicy and inexpediency of organizing separate schools for the specialty of dental surgery—and, secondly, from the incompetency and inefficiency of a part of the faculty of the Baltimore College; that dental colleges so far as they have been tested, have most glaringly magnified an *evil* they profess to correct, and have acted as a greater drawback upon professional advancement and improvement, so decidedly inefficient are they, than if they had never existed, &c.

We think from our acquaintance with the resources, the operations, and the results of the first regularly organized dental college, extending now over a period of twelve years, that we are in a position to furnish a *rather* more reliable account of the utility, and practical working of the scheme, than can reasonably be expected from individuals whose information appears, from their writings, to be extremely limited and meagre; and who, up to the present, have never exhibited a very deep concern, or a very lively interest in *any* plan or scheme that looked to the elevation and improvement of dental science. Indeed, we have been not a little amused, at the new-born zeal of two gentlemen, who have perhaps grown gray in the profession, without ever before having given signal proofs of their devotion to its interests. Where is the record or evidence that these gentlemen have ever before contributed their efforts, or lent their influence, to any measure that looked to the general good of dental science, its cultivation or improvement? We know of none; we do know, however, and those who are familiar with the first organized and efficient effort in that direction, the institution of the American Society of Dental Surgeons, may remember the part played by these neophyte reformers. Then, as now, they acted in concert—they withdrew themselves, and stood aloof from all co-operation with the oldest, the wisest and best members of the profession, who banded themselves together as brothers, and forgetting or losing sight of *self*, honestly and arduously labored for the benefit and promotion of this department of the healing art. But Drs. Gardette and Trenor withdrew themselves, we suppose, to the accomplishment of their private and individual pursuits, and left this benevolent and philanthropic work to be done by other and abler heads and warmer hearts; contenting themselves to be indifferent spectators, if not secret enemies of a revolution in dental surgery, more important and beneficial in its results, than marks any epoch in its history from the days of Ambrose Paré to the present. But lo! "a change comes o'er the spirit of their dreams," suddenly they are transformed from "armed neutrals," to active and zealous champions in the cause of dental education, and the improvement and promotion of dental science.

We regret the necessity for the foregoing commentary; we have no fancy for unpleasant personal allusions, but Drs. Gardette and Trenor have recently assumed a position, as friends and patrons of dental surgery and its interests, to which their former action and bearing give no title, their past history and present professions do not harmonize; and it has been for the purpose of exposing this discrepancy, and unmasking their pretensions that we have imposed upon ourself this task. Neither of the gentlemen have any right to complain, for the injustice, the grossness and indelicacy which distinguish their several articles, shut them out from any claim to special courtesy.

We cannot regard either of the individuals under review, as genuine, disinterested friends of dental science; we cannot do so, because, they are both experienced dentists, they have been long in the profession, and are familiar with its wants, its duties and its requirements; both as sane and rational men, are obliged to be conscious of the wrong which, for a most paltry consideration, they are endeavoring to inflict, in attempting to undo what has cost the labor of years to accomplish, and the best talent in the profession to perfect; and both must know and feel at heart the folly and impracticability of the scheme which they are endeavoring to sustain. No medical board should be influenced by their representations, and no dental student can safely trust to their counsel or advice.

The duties and offices of the dental surgeon, mark him as a character distinct from the practitioner of medicine, or general surgery; and necessity, as well as the dictates of common sense, have defined the sphere of his operations as a separate and distinct profession, and one affording ample scope for the exercise of all the talent and energy ordinarily given to men. The health and integrity of the mouth and its appendages, the care and preservation of the teeth, the correction of their irregularities and deformities, and their replacement by artificial substitutes when they are lost, are offices which require for their satisfactory accomplishment, not only a special and peculiar training, but an assemblage of inherent qualities, mental, moral and physical, not always found, nay, somewhat rarely combined in one and the same individual—qualities not altogether essential to the successful apprehension and practice of medicine, and which are comparatively unimportant to the general surgeon.

The accomplished dentist, must of necessity, be an educated man in the ordinary acceptation of the term; and as the parts upon which he is called to operate, are organized, living and sentient members of the physical organization; members sustaining in health, and disease, the most intimate relations with other organs and tissues making up the general system; he should be taught their origin, their composition, structure, connections and relations, anatomical, physiological and pathological; he should understand chemical attractions and affinities, and be taught the general principles of medicine and surgery, with special *reference* and *application* to the organs and tissues committed to his care. He must possess artistic taste, and a highly cultivated mechanical genius. His eye must readily determine just proportions and unerringly discriminate between light and shades, and his hand must possess a cunning and dexterity, that will be adequate to the most complex and delicate manipulations,—a pretty extensive range of faculties and acquirements, but not greater than the exigencies of dental surgery imperiously demand; and he who masters this range, or who cultivates and develops these resources in a sufficient degree to be accounted respectable, not to say distinguished, will have consumed a period of time, and have performed an amount of labor, greater than is deemed at this time essential, (by the medical boards of this country,) for the honors and responsbilities of medicine and surgery.

We shall now briefly allude to the resources of the Baltimore College for the fulfilment of all the indications of a thorough preparation for the successful practice of dental surgery. And what we have to say of the Baltimore College, we regard as applicable to several similar institutions now in successful operation.

The organization of the Baltimore College consists of five regular chairs or professorships, with a lectureship; and to these chairs the following subjects are assigned: Principles and Practice of Dental Surgery; Anatomy, Physiology and the General Principles of Surgery; Pathology, Therapeutics and Hygiene; Chemistry; and Operative and Mechanical Dental Surgery. The institution is abundantly supplied with accommodations for the practical study of anatomy—not the anatomy "of the head and neck," as the enemies of this school have insinuated, but of the entire system. It is likewise provided with extensive and well appointed laboratories; with a museum of anatomical, physiological and pathological specimens of the most perfect and expensive character, and with an infirmary, well supplied with patients, in which the students are required to practice in all the departments of the science, under the eye and guidance of the professors, having charge of that department. The term of study, and the conditions of graduation are almost identical with those prescribed by the first medical schools of the country. The candidate must be of legal age, of good moral character, must have attended two full courses of lectures, one of which must have been in that school—one course in a regular medical school being regarded as equivalent—or private instruction under a competent teacher, with evidence of four years actual practice; he must defend a thesis, upon some professional topic, and submit to the mode of examination suggested by the American Medical Association, viz. three practicing physicians and surgeons, and three dental surgeons, of education and experience not connected with the institution, are to constitute the board of examiners.

Now, to our mind, this seems to be a course of study which, as enforced and carried out in the Baltimore College, and an array of facilities and appliances, quite sufficient to meet the wants and requirements of the most industrious and ambitious student; and we here "testify to that which we do know," and witness that which we have seen.

We confess, while we claim as much regard for dental science, and as much interest in its character and future standing and improvement, as ever animated the bosoms of either Drs. Gardette or Trenor, that we do *not* perceive, in what important respect, the present system of dental education can be modified with advantage. We can see how the system may progress, in the onward march of successful development; and we confidently believe that it will improve, that it will, at least keep pace with its kindred systems and sciences. We believe, that dental surgery is, and will continue to be, as thoroughly and as successfully taught in the dental colleges as at present organized and constituted, as is, or will be, the science of law in law schools, or divinity in the present theological seminaries; or to go still further, as *is* or will be the science of medicine in the medical schools and universities of the country.

From the heaps of dirt, and the mass of rubbish which have lately been thrown at the Baltimore College, by close scrutiny and skillful treatment, some *few grains* of genuine ore may be extracted. Dr. Trenor, in attempting to show that to properly educate a dental surgeon, he must first be taught in a medical school all the details and minutia of medicine and general surgery, observes, "that the public have nothing to apprehend from a man's understanding his pursuit in life too well." To this sentiment we cordially subscribe. We would not restrict the range of the dentist's inquiries or investigations; we would not curtail or diminish his course of study, or the time requisite for its accomplishment. We certainly have no objection to his attending medical lectures, and graduating to all the honors of medicine and general surgery. We do not object to his being M. D., D. D. or LL. D.; but to be a safe and trustworthy *dentist*, he should likewise desire the D. D. S. And, by-the-by, this last symbol or title, appears to be extremely obnoxious in certain quarters—it appears to be regarded with a degree of jealousy, amounting almost to *hate*, by a certain class of dental practitioners. The public have learned to associate with it, an idea of moral and professional excellence and respectability not always accorded to the M. D. who decorates his parlor or office with his parchment trophy, and points his credulous patrons to its cabalistic characters. Hence we frequently meet in the writings of this class, (hostile though they feel and know themselves to be,) intimations of the injustice and illiberality with which they, the *par excellence* M. D. *dental surgeons*, have been treated by dental colleges; and to weaken and impair the influence of a diploma from those institutions, they very naturally turn their artillery against those schools, rather than their alumni, as a contest with them, before the great arbiter, public opinion, would hardly be advantageous.

But again, we are not inimical to medical instruction in its most comprehensive sense, or to any other department of education; and we are not prepared to deny, that medical students would be benefited, and the interests of humanity promoted, by the addition of a *competent* lecturer on dental physiology and pathology to the medical schools. There is no danger to be apprehended "from a man's understanding his pursuit in life too well," but *there is great danger* to be apprehended from his *not* understanding it sufficiently well. And I take leave here to say, that evidence of qualification for *one* pursuit or profession, though it be the title of M. D., is *no evidence* of fitness or capacity for the duties and offices of another and distinct pursuit or profession; and than this, there is no fact more frequently or strikingly illustrated in the practice of dental surgery.

There are, we know, in this wide world a few individuals, who have the wonderful faculty of readily comprehending and acquiring any and every species of knowledge to which they may apply themselves; they become with little apparent effort, and in an incredibly brief period, not only respectable, but distinguished in several sciences or departments of knowledge, either of which, would require the prime of youth and manhood in ordinary cases to accomplish; but these are prodigies—are few and far between.

We know also another class, but unlike the former, they are somewhat numerous and *common*, they are distinguished by great vanity and egotism, they entertain a most exalted estimate of their own abilities, and a correspondingly low and contemptuous opinion of the claims and capacities of their fellows; they esteem themselves adequate to the accomplishment of any and every enterprise however difficult, as capable of mastering any, or all of the arts and sciences, and of traveling in each or all, a bow-shot beyond any predecessor—"no pent up Utica confines their powers." They are known in common parlance, as "Jacks at all trades." But it is from neither of the two classes we have depicted, that society is to be furnished with safe and scientific dentists—there are two *few* of the former, and the latter are utterly worthless and hopeless.

We remark again, that the range of dental surgery is sufficiently extensive to occupy, industriously, all the time, which in the brief period of man's existence can be spared to the study and acquisition of an honorable and useful calling; and its offices, and successful pursuit, are sufficiently important and difficult to require and amply compensate the highest degree of talent. The duties of the dental surgeon being distinct, and well defined by custom and common consent, society does not expect or demand at his hands, the practice of medicine or the operations of general surgery, neither is he expected or required to fulfill the functions of accoucher, occulist or aurist; and on the other hand, society has long since discovered, that it was not its interest to demand at the hands of the general practitioner, the simplest dental operation.

The question here narrows itself to two propositions, which are simple, and may be briefly stated—is it better for an individual to enter upon the practice of a profession with a thorough preparation for the duties *of* that profession, with his head and his hands educated with special reference to that pursuit? or that he should assume its responsibilities, with a mind filled with an indistinct and imperfect apprehension of several kindred pursuits? And is it not most wise and most expedient, in seeking preparation for a profession, to apply where that profession is thoroughly and exclusively taught, where it is the leading and main feature, where all other sciences and arts are made tributary to it, and where every resource and facility is provided; or would it be better to apply where the pursuit of your choice is regarded as a secondary, subordinate and incidental appendage to an institution devoted to another and distinct profession?

We admit, nay more, we emphatically maintain, that the general principles of medicine and surgery form the basis and foundation of sound and thorough dental education, and of all safe and successful practice; but the distinguishing difference between medicine and general surgery, and the specialty of dental surgery, educationally and practically considered, *is the application of those principles.* And as a fair illustration of what we mean, no candidate for legal or judicial honors is expected or required, because there is a revelation of perfect and divine law, to master the subtleties and mysteries of theology. Nor do we believe it was adjudged necessary, that Raphael, Reubens, Reynolds or West should serve an apprenticeship to house and sign painting; or that Michael Angelo, Canova, Greenough or Powers should graduate in a marble quarry; or that it was ever considered necessary to the attainment of that skill which constructs and arranges to their proper positions and offices, the delicate mechanism of the chronometer, that the same head and hand should be equally apt and ready in the building of a locomotive engine, or the gearing of a grist mill. Now it will be at once perceived, that there are general principles applying in common to these sciences and arts, principles identical in themselves, but differing radically in their application and results; and it would not be at all more absurd to expect or require a lawyer to be a theologian, a physician, a divine, or a sculptor a stone mason, than to expect or require of a well educated and skillful dental surgeon, equal skill and equal proficiency in medicine and general surgery.

We have shown the provisions, the facilities and the appliances of the Baltimore College of Dental Surgery to teach as thoroughly as may be, the principles of medicine and surgery, and their application to the specialty of dental surgery, as well as those departments which partake neither of a medical or surgical character, but which are peculiar and which must ever distinguish dental science, as a separate and distinct pursuit. Now what is to be gained by the abolition of this school, the dismemberment of its faculty, and the proposed substitute of a lectureship in the medical schools? in what respect has the Baltimore College failed to fulfill the indications for its establishment? how has it acted as a drawback upon the elevation and improvement of dental science? and what evil that it professes to remedy has it so glaringly magnified? Such charges as are embraced in the foregoing interrogatories, have been unblushingly uttered by Drs. Gardette and Trenor, with what cause, and with what truth, we have been at some trouble to ascertain, and with what design, and for what object, we think we shall pretty clearly show.

It is proposed to supersede the college system by the appointment of an adjunct lecturer to the medical colleges. What the precise duties of this lectureship are to be, we are at some loss to imagine; they have not as yet been defined by either Drs. Gardette or Trenor. It would appear a little unreasonable to suppose, however, that a class could be successfully conducted through the necessary course of instruction, to properly qualify them for the duties of medicine, surgery and dentistry, in a period of time, now universally admitted to be too short for medicine alone, and it would be really requiring a great deal at the hands of an adjunct lecturer, to accomplish in that brief space, what Drs. Gardette and Trenor inform us, the five professors and one demonstrator in the Baltimore College are totally unequal to. We think it would be making a requisition upon the energies and talents of students, to which *few* would respond; as well as imposing a burden upon the lecturer on dental science, to which but two or three individuals of the present age, would be likely to consider themselves equal.

Drs. Gardette and Trenor, have broadly asserted, that the five professors in the Baltimore College, have, after twelve years experience, failed to teach dental surgery in a thorough and satisfactory manner, and that, too, under circumstances and surroundings confessedly favorable. Now, how is it, we beg to know, that one lecturer in a medical college can, in the same time, instruct so much more successfully in dental surgery, a class of medical students, occupied with all the details and departments of medicine and surgery, than can *six* experienced teachers in a dental school? What magic is there in the halls of a medical school that would render one lecturer with qualities and attributes superior to six in a dental school? And what charm is there, that would make Professor Harris, Professor Townsend or Professor Taylor more satisfactory teachers in a medical school than they are in their present positions? We dont exactly mean to join issue with Drs. Gardette and Trenor here, the thing may possibly be accomplished; there might possibly be found two or three lecturers in the world, who would essay, and after a fashion accomplish this feat; but even admitting this, we have no special evidence that these wonderful teachers are destined, through all time, to inhabit this mundane sphere; we know that the good, the great, and the excellent of the earth, are after a season called away, and where should we look for Elishas to receive their mantles?

But Dr. Trenor asserts, that dental surgery is very imperfectly and inefficiently taught in the dental colleges, and that the medical colleges ought to take the subject in hand, and appoint an adjunct lecturer; because nothing is at present taught in these institutions of practical value to the dentist, and both Drs. Gardette and Trenor insist, that the scheme would work to the benefit and advantage of all schools that would adopt their suggestions; that is, that the adjunct lecturer would draw larger classes, we infer. So then, after all, it is the dental professor, upon whom dental students must rely for instruction in dental science; and that professor is no where so competent to impart instruction, as in the halls of a medical college. So much the more increased are his capacities, and enlarged are his faculties, that *one* lecturer may assume the charge of half a dozen departments; and make much better dentists of a medical class, than as many lecturers could in a dental college. This all may be so—but somehow, we cannot unriddle or understand it; that schools in which little is taught relating to dental surgery, and that little of no practical value to the dentist, can be transformed into establishments so much better suited to the education of dental students, by the simple process of appointing *one* lecturer, than are the dental colleges with six medically and surgically educated professors, aided by every means and appliance that the industry, the wisdom and the benevolence of man can command; it is an enigma, the solution of which, we shall leave to the discerning and astute minds of the authors of this enterprise.

But, in sober truth, *nothing* can be gained by the proposed change that will conduce to the dignity or usefulness of dental surgery, nothing that would contribute to the interest or importance of medical schools. Medical students, would as soon consume their time and money in listening to lectures upon astronomy, geology or conchology, as upon a science in which they, for the most part, feel as little interest, and the duties and practice of which, (with the single exception of extracting teeth,) there is as little probability of their engaging in. So far from such an appendage operating as an attraction, if the ticket should be adopted as a regular part of the course in medicine, (and that would of necessity be done, to give it any respectability,) it would drive off fifty students where it would secure one; it would be deservedly regarded as a catch-penny appendage, that would lessen the dignity and character of any medical school adopting it. It would add nothing to the resources of dental science, and give no caste or standing to any student silly enough to avail himself of its provisions. All that can rationally be anticipated from such a scheme, would be the gratification of a little personal spleen, and the fostering of a petty personal ambition.

Dr. Gardette tells us that dental colleges "have been tried, and that the experiment has proven a failure;" and Dr. Trenor asseverates, that they,(dental colleges,)"profess to remedy an evil, which they most effectually and glaringly magnify, they hold out the idea of giving a complete and finished course of instruction on dentistry, while full two-thirds of what should be taught, and that the most important too, viz. *all* the instruction which every medical school inculcates in medicine and surgery, it does not enter into their arrangements, nor do they possess the ability with any degree of usefulness or benefit to perform."

Passing over the elegant and gramatical construction of the preceding paragraph, we will only observe, that it contains broad assertions, unsustained by evidence or argument. Let us examine them. Since when was it discovered, that the Baltimore College had failed? It was organized and put in operation twelve years ago; and despite the hostility of the characters who now assail the system, and the doubts which hung over an untried experiment, it has been sustained, it still survives; each succeeding year increases its claims and hold upon public confidence and esteem; and each succeeding lecture term, shows a steady increase in the number of students; its graduates numbering now over one hundred, constitute an important part of the profession. They are scattered through the length and breadth of this continent, and some of them are filling important and responsible stations in Europe. Many of them have succeeded to extensive and lucrative practice in our large cities; where they have to sustain competition with the most skillful and experienced dentists of the union. And while *all* are regarded with jealous hostility by a certain class, and every feature in their practice, or writings is scrutinized with a view to personal and educational disparagement—what single fact has ever been elicited, to show that the system by which they were educated was defective, or that it was inefficiently and inadequately carried out in the Baltimore College? We challenge Dr. Gardette, Dr. Trenor and *all* who are interested, to point to *one* fact, or one incident, which has ever transpired in the practice of the hundred dental surgeons who have been educated in this school, that can reflect reproach upon their Alma Mater, or create distrust of its capacity for the thorough education of the dentist.

Now, surely, if dental colleges were such perfect and entire failures, if the system was so radically defective, and if their resources were so very inadequate, some *one* at least, most objectionable feature, in the practice of so many graduates, in twelve years time, might and would necessarily have developed itself, and might be readily paraded in evidence by the individuals who are *so exercised* upon the subject of dental education; but no, nothing of the kind has been adduced; all that can be made to appear, is the answer of *one* waggish candidate for graduation, to a very awkwardly constructed question, as to the indications for the judicious extraction of the teeth, and even that bears upon its face, evidence of doubtful authenticity. As the question now stands, dental colleges are a decided failure, and ought to be abated, and an adjunct lecturer on dental science appended to the medical schools, *because*, Dr. Gardette and one or two others have so asserted, and so most ardently desire.

It appears, that Dr. Gardette has allowed himself some slight opportunities of investigating and examining the features and peculiarities of the Baltimore College; he has honored that institution with as many as two visits since its organization; amazing condescension! It also appears that in the plenitude of his benignity, and in the exuberance of his kindness, he was, on those occasions, betrayed into expressions of admiration and satisfaction of what he saw and heard; the Dr., however, now tells us, that he was insincere in those declarations and expressions; that the nice things he said of the college, the ability of its faculty, and the proficiency of its students, were only the responses of good breeding, to the courtesies and civilities extended to him on those occasions. May not the doctor's recent laudation of medical colleges, and medical and surgical professors, be similar responses, in acknowledgment, or anticipation, of similar courtesies and civilities? But Dr. Gardette saw nothing in, or about the Baltimore school to commend, but every thing to displease and convince him, that dental colleges were not the thing; he saw "highly finished and brightly burnished *metal fillings*," "but he didn't see the operations performed, and he is too old to be deceived by mere external appearances, he could not determine the amount of brains employed in the process." Verily, we too should think it somewhat difficult to deceive a man by mere externals, who exhibits in his own character and intercourse, the striking and radical difference between external appearances, and real sentiments and opinions. We had never before understood, that truth and sincerity were the sacrifices offered by scientific gentlemen upon the altar of hospitality. We did not know, until we had the great happiness of perusing Dr. Gardette's articles, that a professional man was expected to suppress his opinions, and to compromit his principles and feelings, in acknowledgment of *any* civility or courtesy.

But let us ask Dr. Gardette, if he had the pleasure of inspecting any "amalgam fillings" while making his reconnoissance of the Baltimore College? they too are "metal fillings," though rarely susceptible of a very high finish. The doctor tells us, also, that he saw specimens of "artificial teeth," but as they were not applied, or to be worn, he could not regard them in any higher light than the productions of any common jeweler or goldsmith. And in another place he remarks, that it would make no particular difference, and be no great descent if this department of dental surgery were entirely committed to such craftsmen. But, tell us doctor, did you see any tusks of the sea horse, or grinders of the sperm whale, in blocks, sections and fragments, garnished with trophies from the dead house or potters'-field? Perhaps, however, if you had, your convictions might have remained unchanged; for you could very properly have referred all such specimens to the ivory turner and the resurrectionist, and have considered it no very great descent, to commit this feature in dental practice, to *their* hands.

But what evil is this, let us inquire, which Dr. Trenor so gravely tells us, the dental colleges profess to remedy; and yet so glaringly magnify? And what two-thirds of all the instruction that every medical college inculcates in medicine and surgery, so important to the dental student; is it that the dental colleges have no arrangements or capacity for teaching? The faculty of the Baltimore College are all graduates of medical colleges, are all M. D's, and the fair inference is, that they have learned all that is taught in those schools—the all important "two-thirds" and the other third beside; and why is it that this indispensable and all sufficient two-thirds of medicine and surgery should not enter into the arrangements, and be thoroughly taught by these professors in a dental college? indeed, several of the professors in the Baltimore Dental School, have been acceptable, not to say distinguished, professors in various medical schools of the country; and why is it, that these same gentlemen should become so incompetent and inefficient when transferred to a dental school?

There is a slight inconsistency in the advantages which Dr. Trenor claims for the dental student in medical colleges, and in what he subsequently says of those institutions in their relations to this specialty, that we confess our inability to reconcile. Remember, Dr. Trenor charges, that full two-thirds of all the instruction inculcated by medical faculties in medicine and surgery, and that too the most important part to the dental student, is not arranged for in dental colleges, nor have the professors in those institutions the capacity to teach it with any degree of usefulness or benefit. Now compare the foregoing with a literal quotation from the same article: "That in all the medical colleges of this country, there is not *one* it is believed, in which, either the professors of medicine or surgery, ever make the slightest allusion in their lectures to this specialty, (dental surgery,) the professors of anatomy being the only ones who touch at all upon the topic, and for all *practical purposes* the *little they do say*, (the italics are our own,) *is altogether useless; in fact it must be considered as totally neglected.*" Where is the all important two-thirds, that no professor in a dental college is capable of teaching? Strikingly fit and proper places these medical colleges for the education of dental surgeons; scarcely an allusion is made to the specialty of dental surgery, and the little that is said is practically altogether useless.

From the exhibition of medical and surgical knowledge which distinguishes Dr. Trenor's article, it is to be hoped that the school in which he now has his honors, and from which he derived his M. D. can boast some more creditable representatives of its teachings, or we shall begin to conclude, that dental colleges are not the only institutions in the country which need remodeling, and which fail to teach full two-thirds of what is most important for a medically educated man to know.

This feature in Dr. Trenor's article (his exhibit of medical and surgical proficiency) is peculiarly unfortunate in its bearing upon the object he evidently contemplates; it exposes the shallowness and emptiness of his pretensions as an educated man, to a melancholy extent, and indicates his utter unfitness and incapacity for the office of a professorship in any school of science. Dr. Gardette has, in this particular, shown far more discretion; he has wisely, for himself, refrained from imitating this example, and making an expose of his attainments in those departments of learning so inefficiently taught in dental colleges, and yet so important to the dental surgeon. Nevertheless, he pronounces Dr. Trenor's production "*able*," whether he really so esteems it, whether it is only the "response of good breeding," to an acceptable service; or whether it is a sly and artful expression of irony, we cannot precisely make out.

It would be amusing, could we follow Dr. Trenor through his illustrative cases, in which he stultifies the practitioners of medicine and general surgery, and most successfully controverts, and upsets all the claims and positions, which he urges, and assumes in behalf of medical colleges, and their relation to the specialty of dental surgery—in which, the accuracy of his diagnosis, the wonderful certainty of his prognosis, and the readiness and fertility of his resources, are made to place him in such favorable contrast, with medically and surgically educated practitioners, as well as with mere dentists. The magnitude and importance of the cases, their marvelous obscurity, the amount of science and skill ineffectually applied to their treatment; and the promptness with which their true characters were revealed, and their progress attacked and overcome by Dr. John Trenor, is most amazing. All who would enjoy a hearty laugh, may find food for merriment, in this expose of empty pretension and magnificent folly.

Dr. Trenor draws from his reported cases the following inferences: first, the inability of the medical practitioner, aided though he be by the mere mechanical dentist, fully to comprehend or successfully to treat diseases of such and similar character. Secondly, that there are indications and functions to be fulfilled in the specialty of dental surgery, "which more correctly come within the sphere of the educated dentist, but he must be fully instructed in all the requisites of his department. The practitioner of medicine is obviously incompetent to their management." Thirdly, "the incapacity of the mere mechanical dentist even to comprehend, much less to prescribe, understandingly, for the treatment of diseases on which he is nevertheless constantly consulted." We wish to know in the first place, if the medically educated practitioner is unable, even with the aid of the mechanical dentist, fully to comprehend, or successfully to treat diseases, of "such and similar character, why it is, that Dr. Trenor insists so pertinaciously, that there is no place like a medical college, and no teachers comparable to the professors in those colleges, as preceptors for dental students." We do not suppose, with all Dr. Trenor's admiration for medical professors, that he considers them competent, because they hold positions in a medical college, to teach, what they cannot even comprehend. And what benefit is the mechanical dentist, or the dental student to derive from the teachings of men, whose inability to apply the principles taught by themselves to the treatment of dental diseases, is made so manifest! The doctor's second deduction is fair and legitimate, there are cases, and functions, and duties, "which more correctly come within the sphere of the educated dentist, and he should be *fully* instructed in all the requisites of his department—and those functions, duties and offices, cover the whole ground and domain of dental surgery—but *how*, and *where* to obtain "the full and requisite instruction in this department," is the question.

There are several positions assumed by the writers under review, and several intimations uttered with respect to the teachers in dental colleges and the authors on dental science, which deserves a passing notice in their connection. Dr. Trenor observes, "while the usefulness of medical knowledge to the practitioner of dentistry is attempted to be maintained by those of weight and authority, at least on most other professional topics, the necessity of surgical knowledge, is very generally conceded. Yet there is no hazard in asserting the impossibility of thorough proficiency in surgery, unaided by, or unaccompanied with, a corresponding degree of medical acquirements. Yet custom, and even authority require, that the dentist should possess the one, while in this case it denies the other." Now we utterly deny that any such absurdity was *ever* "attempted to be maintained" by any one, either high or low in authority; or by any respectable teacher or writer in dental science; certainly no such attempt can, with the slightest semblance of truth, be attributed to any one connected with the Baltimore College. And we *defy* Drs. Gardette, Trenor or any one else, to point to any *customs*, much less *authority*, for any such absurdity, it is one of those malicious inuendos, rather insinuated than expressed against dental colleges, and as reckless of truth as it is of reason.

Drs. Gardette and Trenor, both appear to entertain a very humble opinion of mechanical dentistry; and there is rather an amusing discrepancy in their respective apprehensions of the nature of this department. Dr. Trenor says, "the mechanical aptitude and tact which the most successful operator requires, is that which most persons with ordinary dexterity, and faithful application can readily and certainly attain." Why is it, then, that the proportion of skillful and successful practitioners, is not more than one to ten; even after long years of faithful application! if this mechanical aptitude is so simple, and this manual dexterity so easy of acquisition, why is it, that not more than one in fifty dental operators, (and medically educated ones at that,) attain to sufficient skill, so to fill a carious tooth, under circumstances more than ordinarily unfavorable, in such a manner, as to arrest, and prevent a recurrence of the disease? that this is true, no honest observer of dental practice will, or can deny.

But Dr. Gardette defines the mechanical part of dental surgery, to be the construction and insertion of artificial teeth—quite a limited and contracted department. We suppose the Dr. must regard the construction and application of artificial palates, obturators, and appliances for the correction of irregularities of the natural teeth, as pertaining to the surgical, or possibly to the medical department of dentistry; for he says, "mechanical dentistry or the making of artificial teeth, seems the only branch excluded, of necessity, from medical colleges, and this would not be undergoing any great descent as the Professor (Prof. Harris,) well knows, should it continue chiefly in the goldsmith's shop." This is from Dr. Gardette; the friend, patron, and reformer of dental education and dental practice, in the latter half of the 19th century. We are to infer that goldsmiths are likely to become prominent characters in the new and improved system. Dr. G. asserts that he never contemplated instruction in practical dentistry as forming a feature in his college course; but that it is to be from private teaching, in the offices and laboratories of private preceptors, that this essential part of a dentist's education is to be obtained, and this he thinks the only mode, &c., &c. Now it is well known, that the medical colleges have *always* been accessible to dental students, and that the plan of private instruction, in the offices and laboratories of practitioners, has been in operation ever since dental surgery has been practiced. And why has not the system in the lapse of a century, succeeded in producing fifty names, that history could honor with a passing notice? Why did it not elevate, dignify and ennoble a confessedly useful pursuit? Why was it left for the Baltimore College, and the American Society, aided by other similar institutions, to give, within the last twelve or thirteen years, caste and character to this specialty? And what guarantee have we that the system will work any better, if revived under the auspices and patronage of Drs. Gardette and Trenor?

Dr. Trenor appears to sanction a much wider range to the mechanical department of dentistry; he remarks, "in what more properly constitutes the mechanical department of dentistry, embracing mainly the setting and arranging of artificial teeth in all their details, as well as all operations upon the natural teeth *in situ,*" &c., &c. This is certainly extending the mechanical department, nearly far enough, and really appears to leave but a small fragment to embrace that all-important twothirds which must be acquired nowhere else but in a medical college. We have no idea that Dr. Trenor would for a moment entertain the proposition to entrust *his* mechanical department to the jewelers; for in that event, he would have, instanter, to turn goldsmith himself.

We think we have quite sufficiently exposed the contradictions, the absurdities, and the errors which distinguish this new exhibition of interest in the cause of dental education; it is a movement not indicated by any want, or necessity, which is at present experienced in obtaining a thorough preparation to practice dental surgery. No public expression of feeling or opinion has called for it, and no motive could have originated it, we sincerely believe, but considerations altogether personal to two or three individuals: it is a scheme conceived in wickedness and folly, and if it ever reaches the parturient stage, it will fall, still-born, into the vortex of worthless and unremembered things; it will hardly be honored with a passing notice in that ominous and fearful history which Dr. Gardette intimates is yet to be written.

The Baltimore College has never failed to fulfill most satisfactorily the indications and wants which created it, and no failure has been anticipated, except by those in whose breasts the "wish and hope was father to the thought," it has magnified no evil, it has acted as no drawback to professional excellence and improvement. But this school, and the system which it illustrates, has been sustained, and that triumphantly; under its auspices and influences, dental surgery has been lifted from the slough of empiricism and knavery; it has been cleansed from the pollution of charlatanism, and placed side by side the acknowledged and accredited sister science of medicine and surgery: no graduate blushes to acknowledge his avocation, the title of dentist, acquired in this school, is neither "a by-word or a reproach;" and no physician or surgeon feels that he is sacrificing his dignity, or condesending to unworthy associations, when he interchanges the social and professional amenities of life with an accredited alumnus of this institution. No graduate of this school has ever uttered a murmur or syllable to its disparagement, and nearly one-fifth of these were previously the graduates of some medical colleges, who had, also, tested the system of office instruction, as well as the slow and unsatisfactory teachings of the school of practical experience. Why did these M. D's forego the pleasures of home, and the profits of practice to matriculate in the Baltimore College, and attend upon its teachings? was it for nothing? And if the system was defective, and the faculty incompetent, would the discovery not have been made, and the fact spread abroad by this class of students? they were certainly as capable of judging in the premises, as either Dr. Gardette or Dr. Trenor, they were M. D's; and some of them dentists, who had been long in practice, and has a whisper of dissatisfaction or disapprobation ever been heard from one of them? No medical man of character and consequence among his fellows, either in this country or in Europe, has stamped the seal of his distrust or disapproval upon the system, or its development in the Baltimore College.

Under the auspices and influences which instituted the Baltimore College, has grown up, and been developed, a literature, which will compare favorably with the current literature of the kindred sciences; the slanderous aspersions of Dr .Gardette to the contrary notwithstanding; all the standard works of European authors have been translated and republished; and our own authors and contributors to the periodical publications, have, in the same time, essentially improved and remodeled the practice of dental surgery throughout the world.

American dentists are now in request in all the great commercial cities of Europe, and some of them are monopolizing the practice of the polished courts of the old world. And yet Drs. Gardette and Trenor, can see nothing in all this, but the means of lending influence to names but little known, with one exception, (Prof. H. H. Hayden,) to the profession—the exercise of arbitrary power, the cunning exhibition of mercenary strategy; and evidence of the decline of dental surgery.

By-the-by, how happens it, that Dr. Gardette, never discovered, or complimented, Dr. Hayden's distinguished character, until he was removed beyond the pale of human jealousy, and human envy? why did we not see this distinguished endorsation of his claims, when it might have accomplished some little good to the institution over which he presided? And now the question occurs to us, would it not be doing Dr. Gardette, and Dr. Trenor, a signal service, if Dr. Harris, and some ten or a dozen more of the lights and pillars of dental science, would just consent to die off? Would not these zealous champions catch their mantles, assume their chairs, and sufficiently compensate them for their premature voyage over the styx, by the praise which they would lavish upon *departed worth?* Gentlemen professors, please consider the subject.

Melancholy to relate, the Baltimore school has succeeded, and the result of that success has created the demand for similar institutions in the great west, in the empire state, and even in the athens of American medicine; but up to this time, that demand has not seemed to indicate the slightest necessity for the services of either Dr. Gardette, or Dr. Trenor, in the capacity of teachers or professors, present or prospective. "Like begets like," is a plain maxim, whether it originated in the "illuminated heights of Lord Bacon's abstract wisdom," or whether it claims some humbler parentage and birth-place, we do not know, but certain it is, we see its truthfulness illustrated in a variety of forms, amid the busy and restless scenes and purposes of life. Dental colleges have given no special evidence of admiration for either of the individuals under review, and they, in turn, most cordially hate, and thoroughly despise dental colleges, "but the truth must be told," a state of "*sullen, fretful isolation*," is hard to be borne; and first, we have a "memorial" to medical trustees and faculties, to establish a dental lectureship in *their* schools; Dr. Gardette, says, "the learning, the industry, and the eloquence, would be the same in the University of Pennsylvania, as in the Baltimore College of Dental Surgery; and furthermore, that he, Dr. G., as well as Dr. Harris, has done some service in the march of improvement, and that there are several other names which he could mention, of past and present times, (we suppose Dr. John Trenor, is one of these,) who have done honor to their profession, and the communities of which they were members, without having enjoyed the advantages of instruction in dental colleges. In all that part of the foregoing which relates to Dr. Harris, and others, who might be mentioned of *past* and *present* times, we have nothing to say, with two exceptions. But pray! what service is this which either Dr. Gardette, or Dr. Trenor, has rendered the march of improvement? And what principle, invention, or original application, is science indebted to either of the gentlemen for?

After Dr. Gardette's memorial had gone forth to fullfil its mission, and its recommendations, its suggestions, and provisions had been analyzed; its errors and evil tendencies pointed out, and its folly and absurdity made manifest by a brief editorial in the American Journal, there came a rejoinder, in the form of an assault upon the Baltimore College, surcharged with misrepresentation, overflowing with splenetic resentment, and abounding in the most unjust and unmanly comparisons, and allusions, and Dr. Trenor, rushes to the rescue, armed with a display of science, learning, and an array of cases to match, truly appalling. He, too, abuses dental colleges without stint or measure, except the measure with which he metes out his fulsome laudation of medical colleges. And the next intelligence we receive from the scene of conflict, is, that the trustees of the New York College of Physicians and Surgeons, have under advisement the establishment of an adjunct lectureship on dentistry, with a view to the appointment of Dr. John Trenor, thereto, of course; who so competent to execute a system, as its inventor and advocate? And how long shall we wait for a similar announcement from some one of the Philadelphia medical institutions?

We may be mistaken in our estimate of the caution, and circumspection, which ordinarily marks the action of medical boards and faculties; some school may be betrayed into the proposed experiment. By great obstetrical perseverance, the bantling may be delivered alive; but it will require all the skill of the surgeons to correct its deformities; and all the science and care of the physicians, to preserve it through a brief, and bootless existence, for, in no event, can it survive the period of first dentition.

